# Engineered Nickel Oxide Nanoparticle Causes Substantial Physicochemical Perturbation in Plants

**DOI:** 10.3389/fchem.2017.00092

**Published:** 2017-11-08

**Authors:** Indrani Manna, Maumita Bandyopadhyay

**Affiliations:** Department of Botany, Center of Advanced Study, UCSTA, University of Calcutta, Kolkata, India

**Keywords:** nickel oxide nanoparticle, genotoxicity, cytotoxicity, antioxidant enzymes, chromosomal aberrations, mitotic indices, environmental hazard

## Abstract

Concentration of engineered nickel oxide nanoparticle (NiO-NP) in nature is on the rise, owing to large scale industrial uses, which have accreted the scope of its exposure to plants, the primary producers of the ecosystem. Though an essential micronutrient for the animal system, supported by numerous studies confirming its toxicity at higher dosages, nickel oxide is graded as a human carcinogen by WHO. A few studies do depict toxicity and bioaccumulation of nickel in plants; however, interaction of NiO-NP with plants is not well-elucidated. It is known that exposure to NiO-NP can incite stress response, leading to cytotoxicity and growth retardation in some plants, but a defined work on the intricate physicochemical cellular responses and genotoxic challenges is wanting. The present study was planned to explore cytotoxicity of NiO-NP in the model plant, *Allium cepa* L., its internalization in the tissue and concomitant furore created in the antioxidant enzyme system of the plant. The prospect of the NiO-NP causing genotoxicity was also investigated. Detailed assessments biochemical profiles and genotoxicity potential of NiO-NP on *A. cepa* L. was performed and extended to four of its closest economically important relatives, *Allium sativum* L., *Allium schoenoprasum* L., *Allium porrum* L., and *Allium fistulosum* L. Growing root tips were treated with seven different concentrations of NiO-NP suspension (10–500 mg L^−1^), with deionised distilled water as negative control and 0.4 mM EMS solution as positive control. Study of genotoxic endpoints, like, mitotic indices (MI), chromosomal aberrations (CAs), and chromosome breaks confirmed NiO-NP induced genotoxicity in plants, even at a very low dose (10 mg L^−1^). That NiO-NP also perturbs biochemical homeostasis, disrupting normal physiology of the cell, was confirmed through changes in state of lipid peroxidation malonaldehyde (MDA), as well as, in oxidation marker enzymes, like catalase (CAT), super oxide dismutase (SOD), and guiacol peroxidase (POD) activities. It was evident that increase in NiO-NP concentration led to decrease in MIs in all the study materials, concomitant with a spike of stress-alleviating, antioxidant enzymes-CAT, POD, SOD, and significant increase in MDA formation. Hence, it can be confirmed that NiO-NP should be treated as an environmental hazard.

## Introduction

Widespread use of engineered nanoparticles (ENPs) in consumer products has given way to their release and accumulation in the environment, where they are readily taken up by living systems inciting stress (Burklew et al., 2012; Maurer-Jones et al., 2013; Tripathi et al., 2016a). Due to their small sizes (100 nm in at least one dimension) and large surface-area to volume ratio, ENPs have close association with living organisms in their surrounding environment; plants, primary component of any ecosystem, have the most dynamic interactions with them. ENPs are readily internalized and accumulated in plants, affecting their fate and transport in the environment (Ma et al., 2013). Though plant interaction with ENPs predominantly results in phytotoxicity, especially at high concentrations, e.g., in tomato, cabbage, carrot, pea, and lettuce, some reports do indicate that exposure to ENPs also promoted growth in onion and cucumber (Cañas et al., 2008; Stampoulis et al., 2009; Tripathi et al., 2017c). Such apparent differences on the effects of nanoparticles on plants may be attributed to the properties and concentrations of ENPs, the plant species exposed, its age and physiological disposition, exposure time, etc. (Stampoulis et al., 2009). Soil-borne ENPs are taken up by plant roots and transported to shoots through vascular systems, depending upon the composition, shape, size of ENPs as well as the plant's anatomy (Lin and Xing, 2008; Wild and Jones, 2009; Lin et al., 2010; Tripathi et al., 2017b). The ability of ENPs to enter plant cells, by endocytosis or non-endocytic penetration, and travel through the nuclear envelop has evoked interest toward the possible genotoxicity of ENPs (Lin and Xing, 2008; Karlsson, 2010). Interaction of ENPs with the plant system may correspond to one or more of the following: (a) chemical effects, when target organisms are exposed to metal ions in solution perturbing ionic balance; (b) mechanical effects, owing to shape, size, charge, valency, and defined interfaces; (c) catalytic effects, on redox reaction or as chelators, (d) binding with macromolecules, like DNA or proteins either by non-covalent or covalent mechanisms; and (e) generating intracellular oxidative stress (Dietz and Herth, 2011). One or more of these interactions result in damaging the intracellular machineries, affecting different aspects of photosynthesis, namely, the electron transport chain and light harvesting complex (Tripathi et al., 2016b), down-regulating the glutathione cycle, compromising the stress alleviating mechanism of a cell (Tripathi et al., 2017a), affecting mitochondrion, and ultimately instigating the cell death pathways (Faisal et al., 2016). DNA damage is presumed to be induced either due to direct intercalation of ENPs or their physical and/or electrochemical interaction with DNA, as well as, ROS-mediated damage (Atha et al., 2012).

Metal ions, belonging to the group comprising of Cu, Cd, Hg, Ni, and Zn, induce toxicity in living systems by binding to crucial cell components, such as, DNA or the sulfhydryl, carboxyl, or imidazole groups of proteins, thereby modifying their activities (Rico et al., 2011). Nickel, the 24th most abundant element in the Earth's crust, occurs naturally in soil and surface water, but anthropogenic activities have contributed to the increase in environmental concentration of nickel (Rathor et al., 2014). A transition metal, Nickel, exists in five oxidation states, which determine its toxicity (Muñoz and Costa, 2012). Nickel is being widely used in the industry, for production of stainless steels, as catalysts in its salt forms, for electroplating, in nickel-cadmium batteries, coins, and electronic products (IARC, 1990; Easton, 1992), plant based nanocomposites (Gogoi et al., 2015). Concomitantly, Ni pollution has caught fancy of researchers, who have reported instances from India and across the world (Zarcinas et al., 2004; Zhao et al., 2008). In recent times, engineered metallic nickel nanoparticles find used as potential catalysts for CO_2_ capture technologies and mineralization processes (Bhaduri and Siller, 2013). Nickel oxide nanoparticles (NiO-NPs) also possess unique properties, which are exploited in industrial products promoting innovative applications, predominantly in electronic devices and as catalysts as well (Song et al., 2008). As their usage escalated, NiO-NP has leached into the ecosystems, finding their way into terrestrial, estuarine, freshwater, and marine ecosystems, with industrial or domestic waste-water output and by aerial deposition (Wiesner et al., 2006; Baker et al., 2014). Exposure to high levels of elemental nickel produces pathological effects in humans and vertebrate models, such as, embryo deformation, contact dermatitis, lung fibrosis, cardiovascular and kidney diseases, and cancer (Ispas et al., 2009; Huang et al., 2011; Coman et al., 2013). In plants, such metal exposure causes interference with cellular processes leading to redox imbalances and oxidative stress (Schützendübel and Polle, 2002; Sharma and Dietz, 2009). Disturbance of mineral nutrition (Parida et al., 2003), photosynthesis (Prasad et al., 2005), water relations, respiration (Llamas and Sanz, 2008) as well as, nitrogen metabolism (Gajewska and Skłodowska, 2009) were reported for plants subjected to Ni stress. Nickel NP, in excess, is toxic for plants, inducing various symptoms of injury, such as, growth inhibition, chlorosis, necrosis, and wilting (Lin and Xing, 2007; Stampoulis et al., 2009). It was reported that NiO-NPs were easily transported into plant systems, inducing both cytotoxic and genotoxic effects (Magaye and Zhao, 2012). Most researchers agree that there exists a dearth in our understanding of how properties of ENPs affect their interaction with the living organisms, though general consensus is that these provoke toxicity probably by inducing oxidative stress, inflammation, immunotoxicity, as well as, genotoxicity (Magdolenova et al., 2014). However, the potential effect on the environment and living organisms therein is still ill-investigated (Nowack and Bucheli, 2007; Ju-Nam and Lead, 2008), and, suitable methodologies to identify interactions of ENPs with cellular components are necessary to achieve a better understanding and determine guidelines for their safe application (Love et al., 2012; Smita et al., 2012).

Toxicity analyses of any chemical necessitate an appropriate *in vivo* or *in vitro* test system. Ecotoxicological perspectives dictate that potential toxicity of ENPs should be studied initially in plants, the autotrophic components of any ecosystem. While, prokaryotic bioassays help in detection of agents capable of inducing gene mutation and primary DNA damages, analyses with eukaryotes enable detection of far greater damage, starting from gene mutations to chromosome damages and aneuploidy (Houk, 1992). Higher plants are most suited as genetic models to assess pollutants, because of their sensitivity to environmental mutagens, as also the possibility of assessing several genetic endpoints, which range from point mutations to chromosome aberrations (CA) (Grant, 1994). Among the higher plants, *Allium cepa, Vicia faba, Zea mays, Tradescantia* sp., *Nicotiana tabacum, Crepis capillaris*, and *Hordeum vulgare* are most extensively used for this purpose (Webster and Davidson, 1969; Grant, 1994). *A. cepa* is regarded as the consistent model to assess chromosome damages and disturbances in the mitotic cycle, due to their large chromosomes and low somatic number (2n = 16). The use of *A. cepa* as a test system was proposed way back in the 1940s for demonstrating disturbances in mitotic spindle with the use of colchicines, and the induction of different chromosomal aberrations (CAs) in meristematic root cells solutions in organic salts solutions (Levan, 1938, 1945; Leme and Marin-Morales, 2009). Ever since, modifications in the *A. cepa* test proposed by numerous workers enabled a more comprehensive assessment of chemicals and pollutants (Fiskesjö, 1985; Grant, 1994; Rank and Nielsen, 1994; Rank, 2003).

In the present study, authors demonstrate the cyto-genotoxic potential of NiO-NP on plants, using *Allium* species, exploring its possible relation to ROS homeostasis perturbation, and, induction of antioxidant defense mechanism *in planta*, linking genotoxicity and oxidative burst mechanism in the root tip cells exposed to different concentrations of NiO-NP suspensions.

## Materials and methods

### Plant materials and growth conditions

Five commercially important species of the family Alliaceae were chosen for this study. Seeds of *A. cepa* (Onion var. Nasik Red), *Allium sativum* (Garlic var. Sutton White), *Allium schoenoprasum* (Chives), *Allium porrum* (Leek var. Prizetaker), and *Allium fistulosum* (Spring Cut Bunching Onion), obtained from Suttons India Pvt. Ltd. These seeds were germinated under controlled conditions and seedlings, of similar age and morphology, were used for subsequent experiments.

### Nanoparticle characterization

Engineered NiO-NP was procured from Sigma Aldrich, (St. Louis, USA) [Product code 637130, molecular weight: 74.69, EC Number: 215-215-7, Pubchem Substance ID 24882831, <50 nm particle size Transmission Electron Microscopy (TEM), 99.8% trace metal basis]. The nanoparticle was directly suspended in deionized, ultrapure water (DI-water), and dispersed by ultrasonic vibration (60 W, 40 kHz) for 45 min to produce seven different concentrations as follows: 10, 25, 50, 62.5, 125, 250, and 500 mg L^−1^.

NiO-NPs were characterized by TEM, Dynamic Light Scattering (DLS), and Zeta (ζ) Potential Measurements. Estimation of morphology and size of NiO-NPs was based on the observations from TEM, performed on a Field Emission Transmission Electron Microscope (JEM-2100F, JEOL, Japan) at 200 keV. DLS was performed on a ZetaSizer-HT (Malvern, UK) to determine the hydrodynamic sizes of the NiO-NPs in suspension. The zeta potential values of NiO-NPs, dispensed in ultrapure water, were determined by Zetasizer 2000 (Malvern Instruments Ltd., UK) as the average of 10 readings and compared to previous reports (Lim et al., 2013). Polydispersity index was calculated as:

$$\begin{array}{l} PDI={\left(\sigma /\text{d}\right)}^{2} \end{array}$$

where σ is standard deviation and d is mean diameter, according to Ates et al. (2016).

### Plant treatment schedule

Seeds were soaked in double distilled water, disinfected and germinated on wet, sterile tissue papers in dark in a growth chamber at optimum temperature (23 ± 1°C). Two to three centimeters of long seedlings with unbranched roots were exposed to different concentrations of NiO-NP suspensions (10–500 mgL^−1^) for 6 h each at room temperature. Each exposure set comprised of at least 15 seedlings, and each set repeated thrice. Double distilled deionised water and 0.4 mM EMS solution were used as negative and positive controls, respectively (Talebi et al., 2012).

### Quantification of nickel in roots of *A. cepa* by ICP-OES

Internalization of NiO-NPs in the root tissue of *A. cepa* exposed to it was studied using ICP-OES (de la Rosa et al., 2004) performed in an inductively coupled plasma-optical emission spectrometer (Parkin Elmer Optima 5300 DV, USA) following a wet digestion protocol (Zhao et al., 1994), keeping the plant tissue in 80% nitric acid at room temperature for a period of 7 days for complete dissolution followed by digestion.

### Determination of root cell viability by TTC assay

Viability of root tip cells was studied in treated and untreated root samples using TTC assay, whereby root tips were incubated with 0.05 M TTC (2,3,5-triphenyltetrazolium chloride) solution in PBS buffer for 30 min. Formation of water-insoluble red formazan was measured at 485 nm (Towill and Mazur, 1975), after incubating the stained roots in 95% ethanol overnight. Percentage of cell survival was calculated from the obtained data according to standard literature:

$$\begin{array}{l} \text{Survival rate }\left(\%\right)=\left({\text{A}}_{\text{t}}-{\text{A}}_{\text{b}}\right)/{\left({\text{A}}_{\text{c}}-{\text{A}}_{\text{b}}\right)}^{*}100 \end{array}$$

where, A_t_ is the average absorbance of test component, A_b_ is the value of blank, and A_c_ is the value of control (Mikula et al., 2006).

### Cytological analyses

#### Orcein squash technique

Twenty-five roots tips from each treatment set of each species were prepared following the conventional squash technique using 2% aceto-orcein according to Sharma and Sharma (1980) and Ghosh et al. (2015). All experiments were repeated thrice.

Orcein stained slides were analyzed under bright field at 1,000x magnification in a Carl Zeiss Microscope (Primostar) for cytological changes, and the mitotic indices (MIs) calculated as the number of dividing cells per number of 1,000 observed cells (Fiskesjö, 1997). The number of aberrant cells per total number of cells scored for each concentration, in all the replicates (Bakare et al., 2000). Cells observed in prophase stage of cell division are excluded from calculations.

#### Fluorescence dye squash technique

Ten roots tips from each treatment set of each species were prepared following the standard protocols as mentioned below. For Acridine-orange staining, root tips were prepared according to the protocol of Pakrashi et al. (2014), with necessary modifications. Stained slides were analyzed under fluorescence light (excitation/emission in nm-502/526 for DNA) at 1,000x magnification under a Leica fluorescence microscope (Model-DMIL LED S-80), in dark, to avoid photo bleaching. Root tips were squashed following staining in DAPI (4′,6-diamidino-2-phenylindole) dye for 15 min (30 mM solution in 1X PBS), and prepared according to the protocol of Yang et al. (1993) with necessary modifications. All the slides were analyzed under fluorescence light (excitation/emission in nm-350/470) at 1,000x magnification in an Olympus Confocal Laser Scanning Microscope (CLSM) (1X CLSM 81). Software version: Flouview FVV 1000, 350–470 filter.

### Determination of lipid peroxidation

To determine lipid peroxidation, Malonaldehyde (MDA) content in the NiO-NP treated root tissue were studied against the untreated roots, and quantified (Heath and Packer, 1968), using the TBA-TCA protocol (Verma and Dubey, 2003). Fresh root samples were crushed in presence of 0.25% TBA in 10% TCA and the mixture was heated to 95°C for 30 min before cooling in an ice-bath, and centrifugation at 10,000 × g. Absorbance of the colored suspension was measured at 532 nm. Concentration of MDA was calculated from acquired data using the extinction coefficient of MDA which is 155 nM^−1^ cm^−1^ at 532 nm.

### Determination of antioxidant enzymes

Antioxidant enzyme activities in the roots, both untreated and treated, were evaluated by studying the enzymes: super oxide dismutase (SOD) (Beauchamp and Fridovich, 1971), Catalase (CAT) (Aebi, 1984), and guaiacol peroxidase (POD) (Hemeda and Klein, 1990). Tissue extract was stored at −20°C after preparation by crushing in 50 mM phosphate buffer (pH-7.4) followed by centrifugation at 10,000 × g. Total protein was calculated in a multiwall microplate reader (Biorad, A600) using Bradford reagent (Bradford, 1976). Enzyme activities were measured spectrophotometrically and subsequently enzyme content per 100 mg tissue were calculated using the particular coefficients for each of the enzymes measured.

### Statistical analysis

MI, CA were scored to calculate MIs, phase indices, and total abnormality percentages (Pakrashi et al., 2014) at different concentrations of nanoparticles suspensions. At least 1,000 cells were scored for each observation, and every experimental set repeated thrice.

The statistical analyses were carried out in MINITAB environment (MINITAB ver. 18). Data were presented as mean ± standard error. The results were subjected to one way analysis of variance (ANOVA). The level of significance was established at *p* > 0.05 at every instance for the cytological and chromosomal studies and for all the biochemical assays, throughout the study. In case, a significant interaction was found among the factors (species × concentrations of NiO-NP), such instances were compared by Tukey's Test and Dunett's Test (Ahmed et al., 2017).

## Results

### Nickel oxide nanoparticles form agglomerates in solution

**Figure 1:**
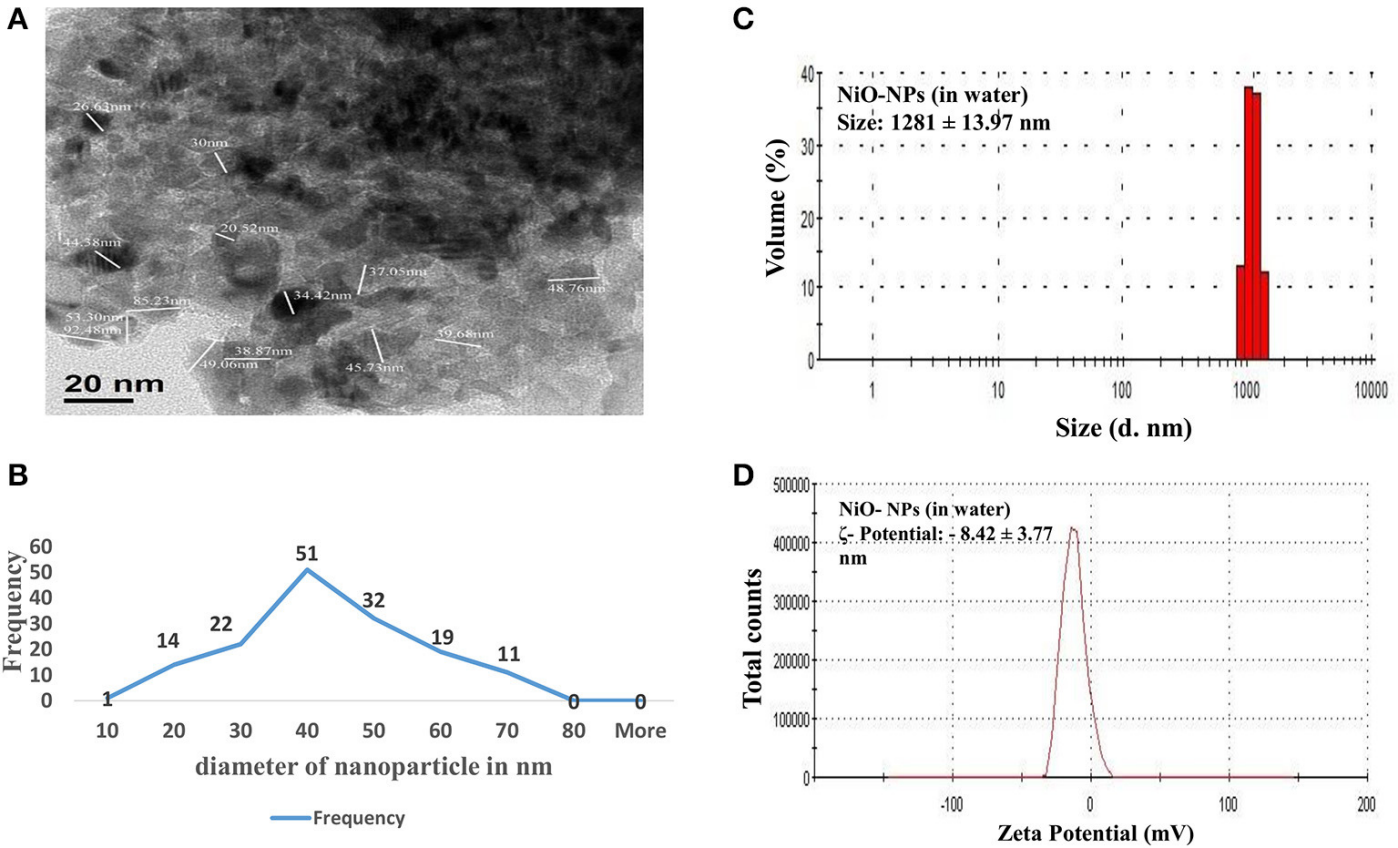
**(A)** TEM image of a NiO-NP; **(B)** Representative graph of particle sizes through TEM imaging NiO-NPs; **(B)** Histogram showing size of NiO-NPs in TEM analysis; **(C)** Dynamic light scattering (DLS) data of NiO-NPs suspension in ultrapure water; **(D)** Zeta potential analysis of NiO-NPs suspension in ultrapure water.

TEM analysis was performed to elucidate and evaluate size, as well as, distribution of NiO-NPs, while DLS analysis helped study the physical parameters in aqueous suspension. TEM analysis revealed that most NiO-NP particles tended to form agglomerates with non-uniform morphology (Figure 1A). The average size calculated from measuring 100 particles in random fields of TEM view (Figure 1B), was found approximately 33.65 nm (size range from 20 to 50 nm) (Figure 1C). Aqueous suspension of NiO-NP studied through DLS showed high rate of agglomeration, as indicated from distribution curves in water (measuring between 1,100 to 1,250 nm) (Figure 1C). The ζ potential of NiO-NP in water was found to be −8.42 ± 3.77 mV (Figure 1D). Contemplating the difference in sizes in TEM and DLS analysis, polydispersity indices (PDI) of NiO-NPs in aqueous suspension for dosage related toxicity analyses were calculated (Table 1). PDI data revealed that aggregation at the higher concentration might be responsible for the aggravated toxicity of the NiO-NP suspensions. All these data of NiO-NP characterization conform to those reported earlier by different authors (Siddiqui et al., 2012; Faisal et al., 2013).

**Table 1:** Polydispersity indices of NiO-NP in aqueous suspension through Dynamic Light Scattering (DLS) mechanism.

**Nickel oxide nanoparticle concentration**	**Polydispersity indices**
10	0.116 ± 0.011
25	0.254 ± 0.054
50	0.38 ± 0.0322
62.5	0.431 ± 0.017
125	0.486 ± 0.048
250	0.540 ± 0.0579
500	0.69 ± 0.033

### Nickel ion is internalized in treated *A. cepa* roots with increasing NiO-NP concentration

**Figure 2:**
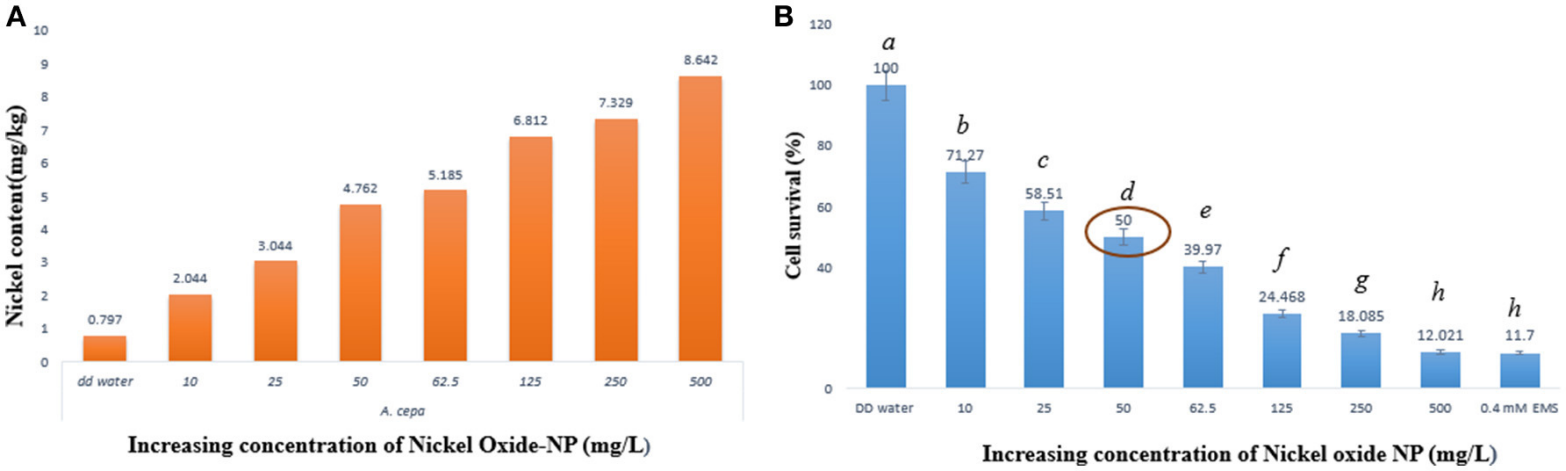
**(A)** ICP-OES result showing content of nickel ion in the treated samples, **(B)** Cell survival percentage calculated through formazan formation.

Concentrations of nickel ions internalized by the treated *A. cepa* root tips were measured using ICP-OES, in comparison to untreated ones (Figure 2A). Untreated samples showed presence of a residual amount of Ni ions (0.797 mg kg^−1^ fresh weight), which was expected since Ni ions are known to function with many important enzymes. However, a linear rise in Ni concentration in the NiO-NP exposed roots indicated direct correlation of the amount of internalization of Ni with increase in concentration of NiO-NP suspension. A 10–11 fold higher Ni content in roots exposed to the highest concentration of NiO-NP with respect to the control set thus provided clinching evidence that NiO-NPs were indeed internalized in the exposed root tips.

### Nickel oxide nanoparticles retarded cell viability and metabolic activity

Cell viability assay was performed using TTC (Ahmed et al., 2017) whereby TTC, a white compound, reacts with dehydrogenases found in living cells to form a red product (TPF–1,3,5-triphenylformazan). This is an important test to prove presence of metabolic active cells, as non-viable cells do not form hydrogenases and as such no formazan, hence no color change can be observed. Interestingly, root cap and meristematic zone of root tips when treated to increasing concentrations of NiO-NP suspensions showed concomitant decrease in color intensity, clearly depicting progressive cell death. Cell survival percentage gradually declined in a dose dependent manner with increasing concentrations of NiO-NP, and < 50% cells survived exposure to NiO-NP concentrations of 50 mgL^−1^ and above (Figure 2B; Supplementary Figure 1). Lowest percentage of surviving cells was observed in roots exposed to the positive control 0.4 mM EMS, which was comparable to the percentage of cell surviving in roots exposed to 500 mgL^−1^ NiO-NP.

### Nickel oxide nanoparticles induced cytological instability

Conventional orcein staining and fluorescent AO and DAPI staining were performed to study the effect of NiO-NP exposure on cell division in root meristems of different *Allium* species.

**Figure 3:**
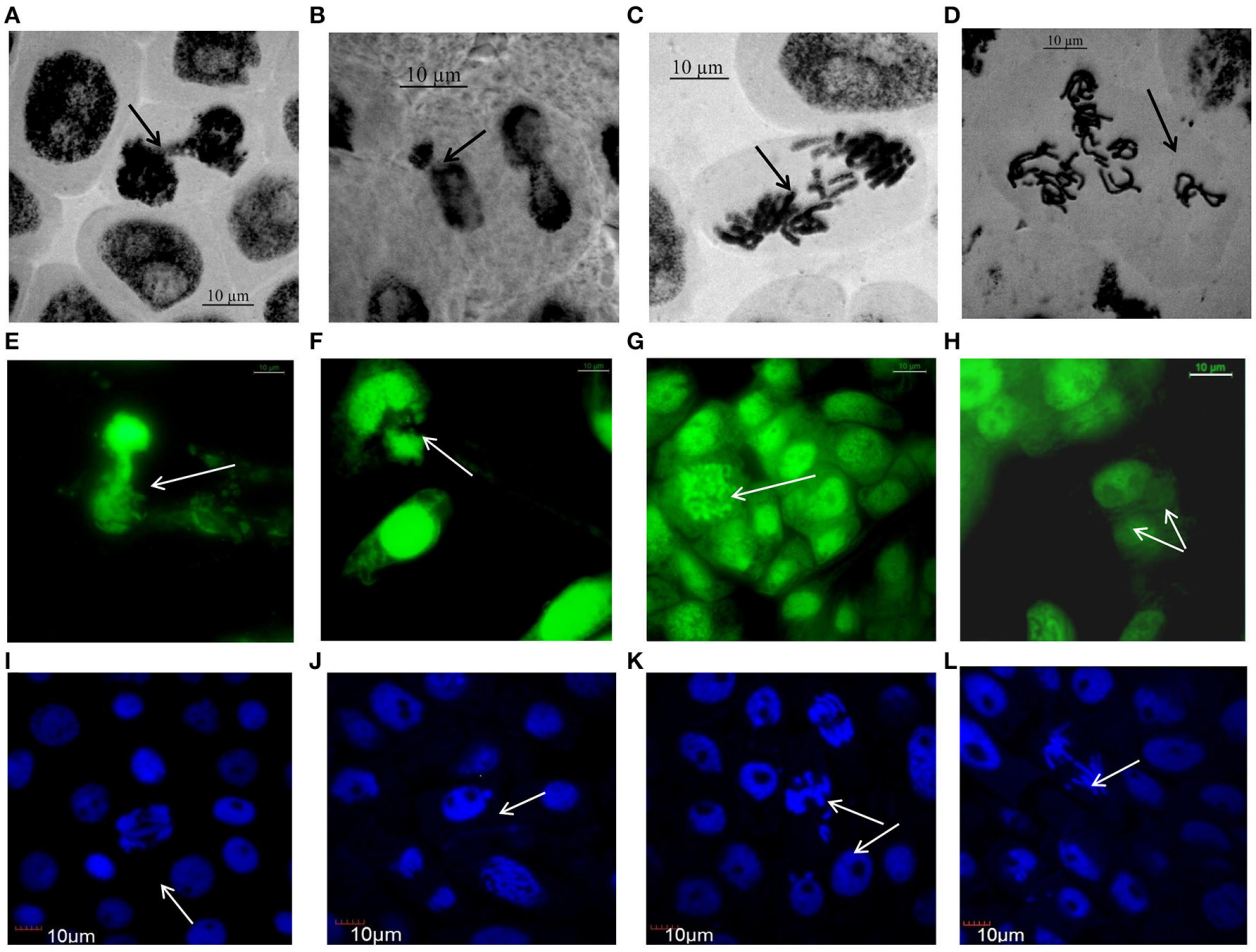
Aberrations induced by NiO-NP suspension in various species of *Allium* as observed under Optical, Flourescene and Confocal microscopes: **(A)** telophase bridge in *A. sativum*, **(B)** nucleus breakage in *A. schoenoprasum*, **(C)** laggards in *A. porrum*, **(D)** vagrants after duplication in *A. cepa*, **(E)** nucleus damage in *A. cepa*, **(F)** chromosome breakage in *A. porrum*, **(G)** C-mitosis in *A. fistulosum*, **(H)** binucleate cell in *A. sativum*, **(I)** double anaphase bridge in *A. cepa*, **(J)** micronucleus in *A. cepa*, **(K)** laggard and vagrant chromosomes in *A. fistulosum*, **(L)** anaphase bridge with laggard and early migration in *A. porrum* (bar = 10 μm).

Figure 3 (Supplementary Figures 2–4) represents compilations of some stages of mitosis in cells of NiO-NP exposed root meristem. Potential toxicity of NiO-NP on test plants was evaluated based on standard cytological parameters like MIs, CAs, and micronuclei (MN) formation. While MI were used in evaluation of cytotoxicity, frequency of CA, and MN formation were used to evaluate genotoxicity.

**Figure 4:**
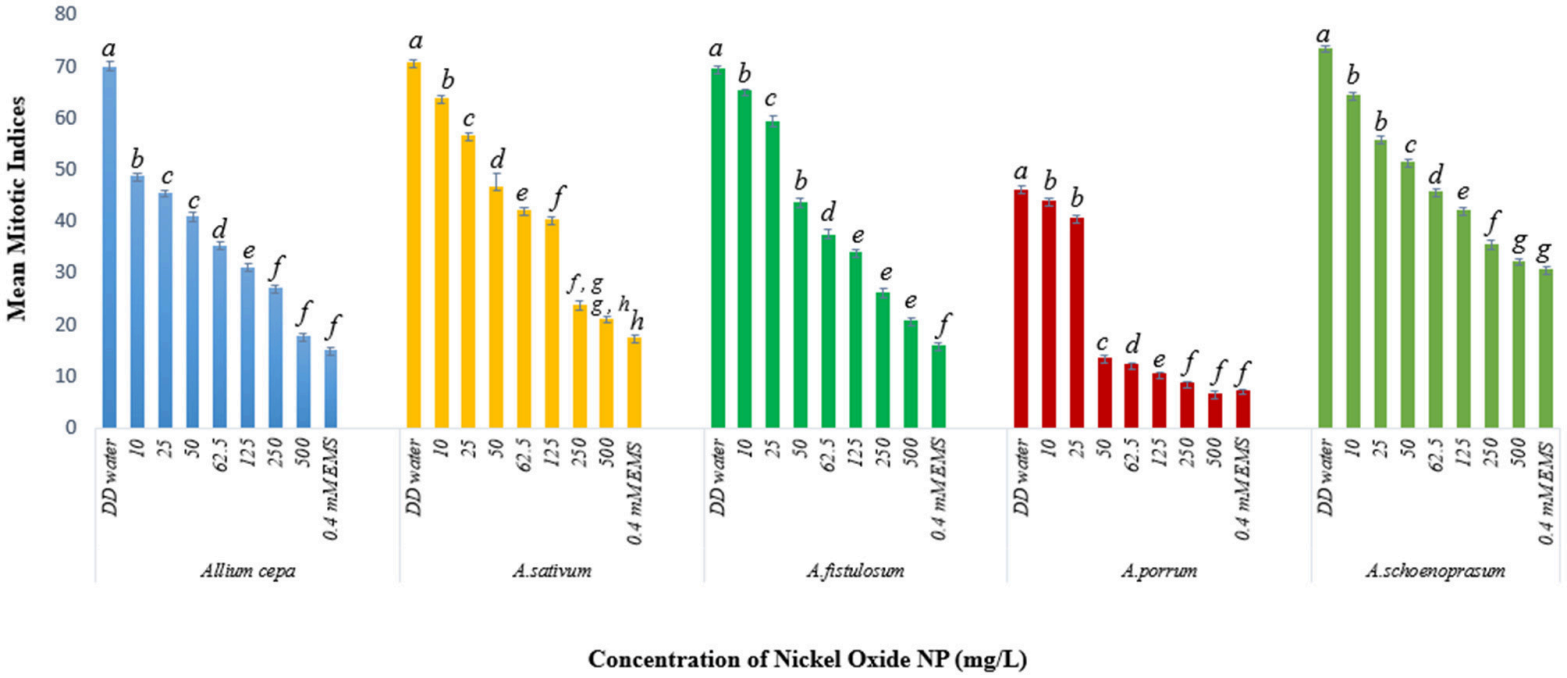
Effect of NiO-NP on mitotic indices in the five species of *Allium* sp. (*p* < 0.05).

Mitotic indices of the treated samples were significantly (*p* < 0.05) altered when compared to the positive and negative controls (Figure 4). For the untreated control plants MIs ranged from 21.5% in *A. fistulosum* to 13.833% in *A. porrum* seedlings germinated in deionised distilled water. Exposure to even the lowest concentration of NiO-NP used (10 mgL^−1^) was shown to decrease the MI significantly (*p* < 0.05) in all the plants studied when compared to the negative control. At the highest NiO-NP concentration (500 mg L^−1^) MI of root tips in all the species studied, were comparable to those observed in presence of the positive control (0.4 mM EMS). In general, MI of exposed root tips decreased gradually with increase in NiO-NP concentration, till MI was severely repressed upon exposure to NiO-NP suspensions above 250 mg L^−1^. When root tips were treated with 0.4 mM EMS solution, a known mutagenic and teratogenic compound used as the positive control, MI was found to be very low in all the plants, ranging from 3.323 ± 0.088% in *A. cepa* to 1.427 ± 0.1% in *A. schoenoprasum*.

**Figure 5:**
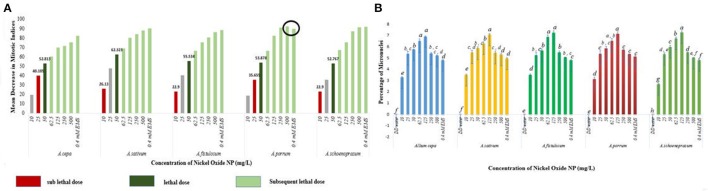
**(A)** NiO-NP suspension being sub lethal to lethal in every concentration in all the five species of *Allium*. In *A. porrum*, 0.4 mM EMS shows to be less toxic than 500 mg L^−1^NiO-NP suspension; sub lethal dose where MI decreases more than 22% of that of negative control, lethal dose where MI decreases more than 50% of that of negative control; **(B)** Percentage of micronuclei formation on NiO-NP exposure (*p* < 0.05).

The stated variations in MI in treated sets as compared to the positive and negative controls were interpreted in context of CLV (Figure 5A, Supplementary Table 1). A decrease of MI below 22% of negative control is deemed to be sublethal and that below 50% is lethal (Sharma and Vig, 2012). This is termed as Cytotoxic Limit Value (CLV) and an assessment of the CLV in each of the five chosen plants was conducted (Figure 5A). In the present study, NiO-NP suspension at 10 mgL^−1^ concentration was found to be sub lethal to *A. sativum, A. fistulosum*, and *A. schoenoprasum* while the concentration of 25 mgL^−1^ was sublethal to the other two species namely, *A. cepa* and *A. porrum*. However, all concentrations from 50 mg L^−1^ onwards were deemed to be lethal in all the species used (Supplementary Table 1; Figure 5A). From the study, it was thus evident that NiO-NP exposure causes cytotoxicity in the treated samples. The effectiveness of ENP suspension in progressive inhibition of MI can be expressed as *A. fistulosum* > *A. schoenoprasum* > *A. sativum* > *A. cepa* > *A. porrum*.

Another important cytological marker for toxicity, MN, were observed predominantly in the root tips exposed to lower concentrations of ENPs (10, 25, 50, 62.5, and 125 mgL^−1^). Frequency of cells with MNs peaked in root tips exposed to 125 mgL^−1^ NiO-NP with highest percentage of MN occurrence found in *A. cepa* (6.93%) and *A. schoenoprasum* (7.31%) and incidence of MNs decreased at higher concentrations (250–500 mgL^−1^) (Figure 5B), probably concomitant with the general scarcity of dividing cells in all the plant species studied (Figure 5B). NiO-NP suspension induced MN formation at even the lowest concentrations, thus indicating potential genotoxicity.

**Figure 6:**
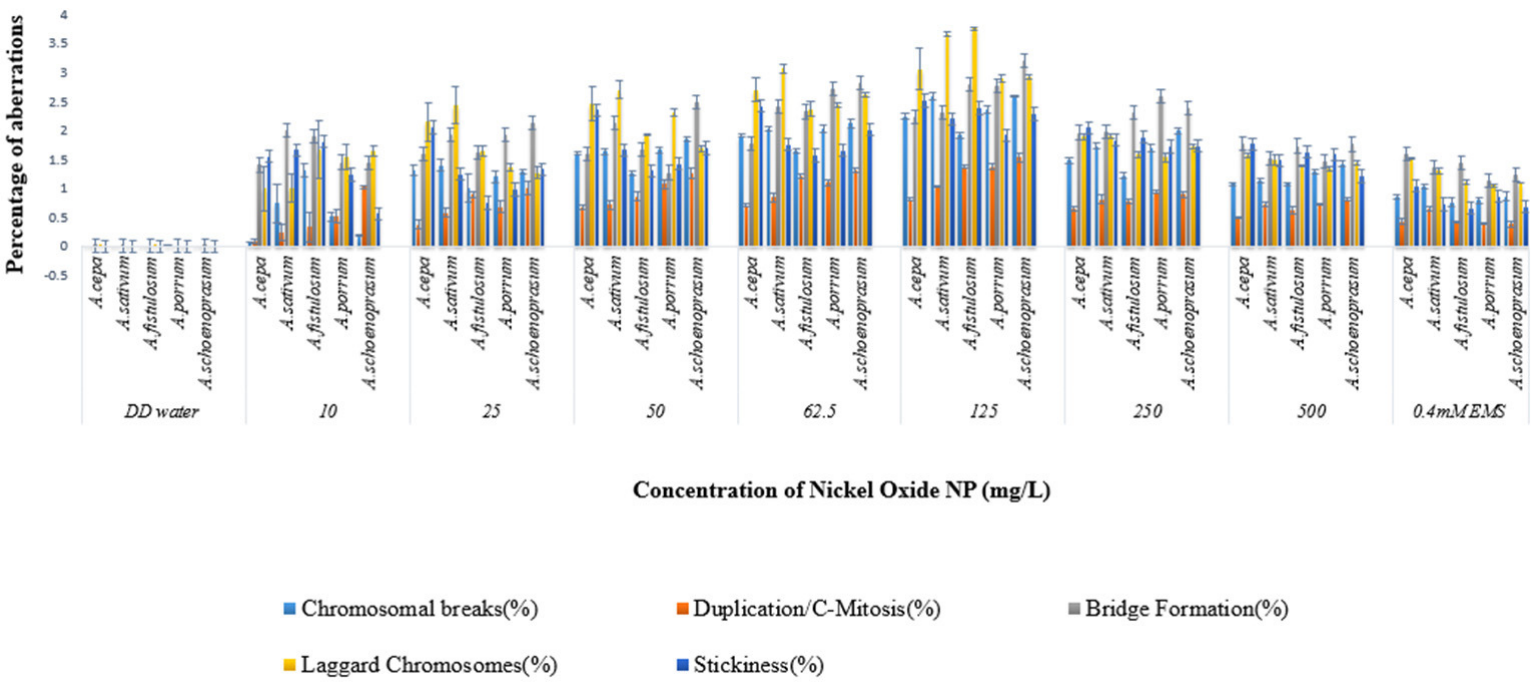
Incidence of various aberrations on NiO-NP exposure (for Anova analysis, consult S.I).

**Figure 7:**
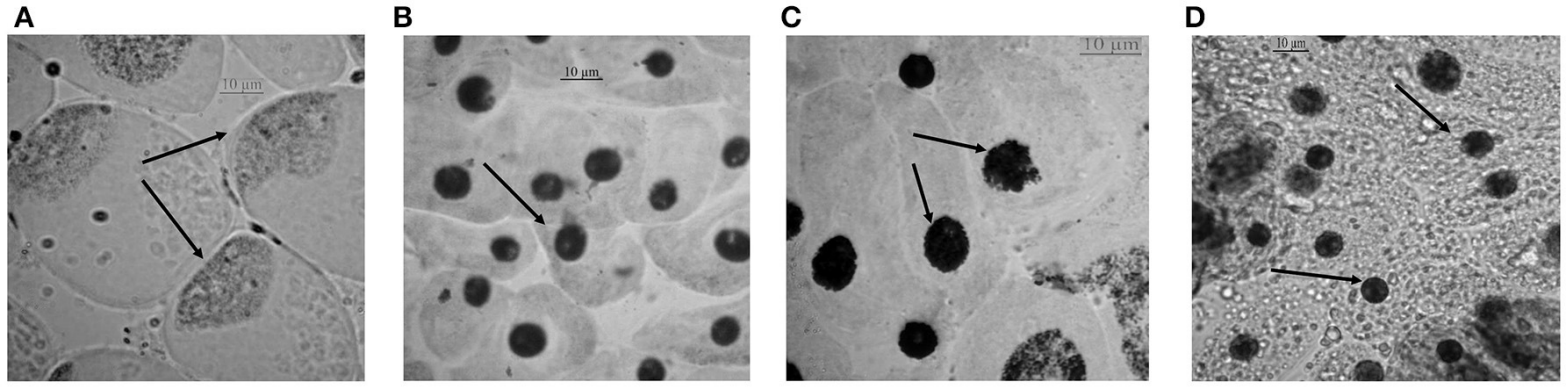
Cells approaching pyknosis: **(A)** condensation of nuclear matter at a corner of the cell in *A. porrum* at 250 mg L^−1^ of NiO-NP suspension; **(B)** condensed nucleus in *A. sativum* at 250 mg L^−1^ of NiO-NP suspension; **(C)** fragmentation and condensation of nucleus in *A. cepa* at 500 mg L^−1^ of NiO-NP suspension; **(D)** pyknosis in *A. fistulosum* at 500 mg L^−1^ of NiO-NP suspension.

**Figure 8:**
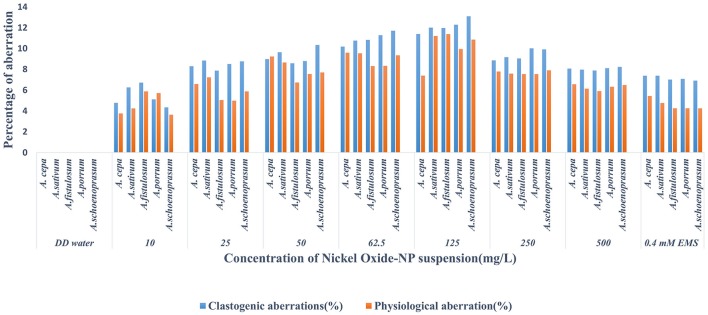
Incidence of Clastogenic and Physiological aberrations on NiO-NP exposure in the five species of *Allium*.

Chromosomal aberrations (sticky bridge formation, laggards, clumped metaphase, chromosome breaks, and binucleate cells) were observed in varying frequency in all stages of mitosis (Figure 6; Supplementary Figures 2–4) in root tips exposed to different concentrations of ENP, anaphasic, and telophasic sticky bridges being most prevalent. The frequency of occurrence of aberrations was found to be dose-dependent and showed a linear increase with increase in ENP concentration to which the roots were exposed (Figure 6; Supplementary Figure 5). The two highest concentrations of NiO-NP used (250 and 500 mg L^−1^), showed very erratic cell division cycles, shrinkage of chromatin and advent of Pyknosis (Jones, 2000) (Figure 7). This observation was found to be in conformity with the observations recorded when root tips of these *Allium* species were exposed to the positive control (0.4 mM EMS solution). Chromosomal aberrations of both clastogenic (MN formation, chromatin bridges, breaks, and rings) and physiological types (C-mitosis, vagrants, stickiness, and laggards) were found in all the plants studied (Figure 8).

### Increased lipid peroxidation indicates membrane damage in roots exposed to NiO-NP

**Figure 9:**
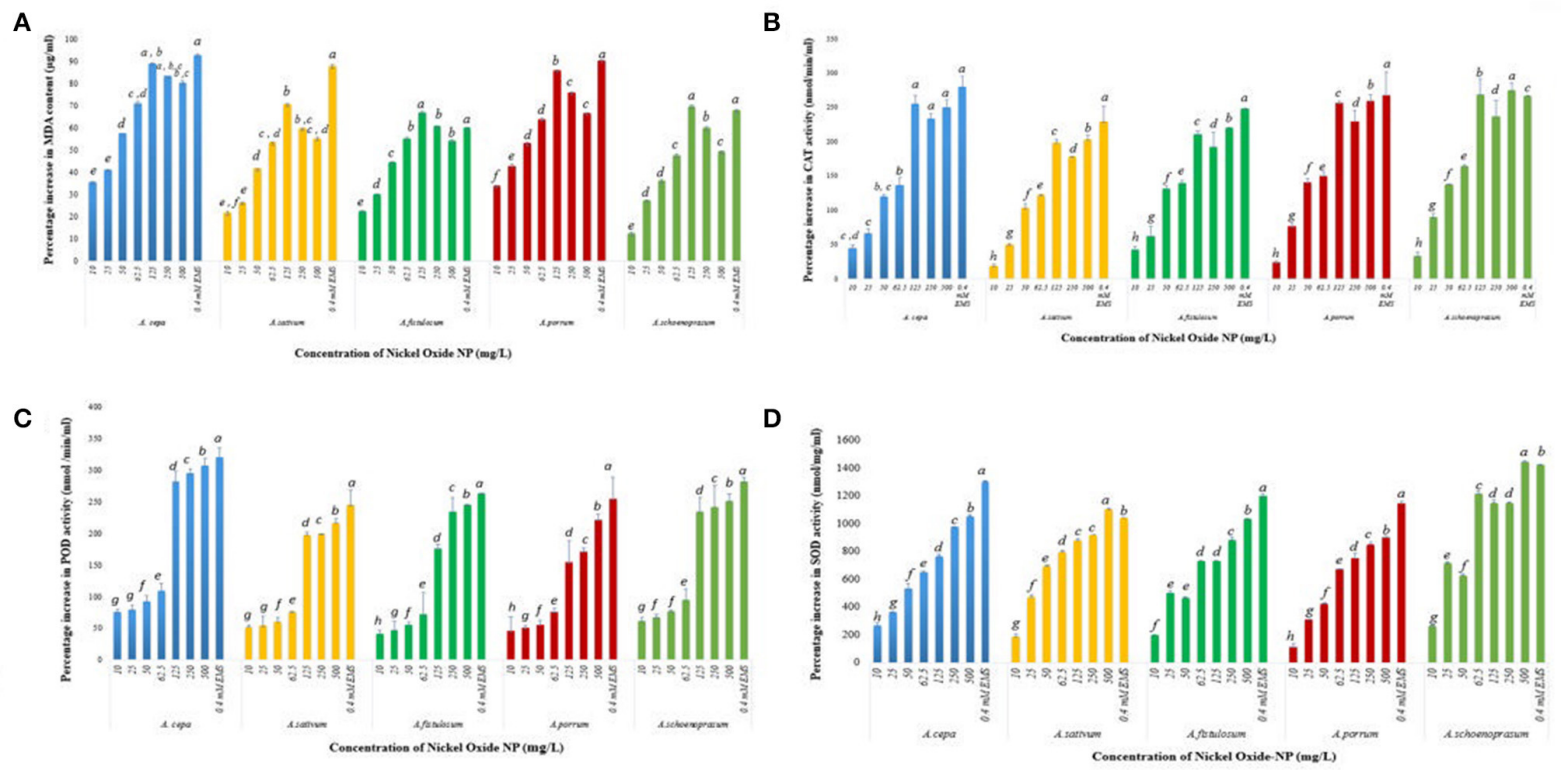
**(A)** Effect of increasing NiO-NP concentration on malonaldehyde content (lipid peroxidation) in the treated samples; **(B)** Effect of increasing NiO-NP concentration on catalase activity; **(C)** Effect of increasing NiO-NP concentration on POD activity; **(D)** Effect of increasing NiO-NP concentration on SOD activity, expressed as percentage change, expressed as percentage change (*p* < 0.0.05).

The membrane lipids undergo peroxidation during incidences of stress, causing their degradation and forming thiobarbituric acid reactive species (TBARS), especially MDA that can degrade DNA (Tuteja et al., 2001). ROS mediated lipid peroxidation is the most well-known pathway (Girotti, 1998) of perturbation of membrane lipids. A basal level of 1.04 ± 0.03 μg/ml MDA was detected in the untreated control roots which spiked upon introduction of NiO-NP in all the species, notably *A. cepa*, where MDA content of roots increased from 1.41 ± 0.04 μg mL^−1^ at 10 mg L^−1^, 1.64 ± 0.01 μg ml^−1^ at 50 mg L^−1^, and highest at 125 mg L^−1^ i.e., 1.97 ± 0.02 μg ml^−1^; decrease in LPO formation was detected in the next two higher doses (250 and 500 mg L^−1^) (Figure 9A). Similar observations were recorded for all the other species of *Allium* studied.

### Phytotoxcity of NiO-NP confirmed by decline in protective antioxidant enzymes profile

NiO-NP exposure was found to alter activities of antioxidant enzymes, thus inducing oxidative stress within the exposed tissues. To examine the amount of oxidative damage inflicted, alteration in profiles of SOD, CAT, and POD enzymes were analyzed in all the treated samples, as well as in both the positive and negative controls. A basal CAT level of 97 ± 1.3 nmol min^−1^ ml^−1^ was observed in the untreated control in *A. cepa*. However, with introduction of NiO-NP treatment CAT activity showed a steep rise with highest activity being recorded at 125 mg L^−1^ concentration (Figure 9B), and, in fact, a decrease in CAT activity was recorded in all the species at the two highest concentrations (250, 500 mg L^−1^) (Figure 9B). In *A. cepa*, the basal CAT level stood at 115.66 ± 1.1 nmol min^−1^ ml^−1^, which rose to 138.022 ± 4.3 nmol min^−1^ ml^−^ at the introduction of NiO-NP (10 mg L^−1^). Such a steep rise in CAT level was observed in the other species too. Changes in POD activity was tracked by the amount of tetraguaiacol formed, detected at 470 nm for a time period of 180 s. Untreated samples of *A. cepa* showed a basal level of 83 ± 0.76 nmol min^−1^ ml^−1^ POD activity, which like CAT, POD also showed higher activity in the treated samples with respect to controls, with highest increase at 125 mg L^−1^ in all the species studied. Further increase in NiO-NP concentrations (Figure 9C), POD activity decreased in *A. sativum* and *A. schoenoprasum*, with minimal increase in *A. cepa* (12%), *A. porrum* (32%), and *A. fistulosum* (11%) increase over 125 mg L^−1^. Similar results were recorded for SOD activity too, there was 236% jump in activity in *A. cepa* at 10 mg L^−1^ NiO-NP over untreated control (from 5.22 ± 1.3 to 19.2 ± 2.3 nmol min^−1^ ml^−1^ in *A. cepa*), around 400% increase in *A. fistulosum, A. sativum*, and *A. schoenoprasum* at the lowest dose; the trend was replicated in all the species studied in all the concentrations. SOD content increased linearly in a dose dependent manner till the highest dose (Figure 9D).

## Discussion

Engineered NiO-NPs have garnered global attention, as one of the major pollutants, owing to its extensive usage in the metallurgical and electronic industries (Gong et al., 2011). Earlier studies have indicated that NiO-NP induces cytotoxicity on cultured human cell lines by stimulating ROS-mediated apoptotic pathways (Siddiqui et al., 2012; Ahamed et al., 2013), warranting extensive studies and safety assessment of this ENP. However, there are not many reports on toxicity assessments of NiO-NP in plants available in the public domain. Potential phytotoxicity of NiO-NP was reported in tomato through induction of oxidative stress mediated apoptosis/necrosis (Faisal et al., 2013). Though these workers did indicate potential interaction of NiO-NP with DNA, there were no assays on genotoxicity to corroborate their claims. The present study focuses on investigation of the potential toxic effects of various concentrations of NiO-NP on *Allium* species. To the best of our knowledge, this is the first in-depth study which shows that nickel oxide NPs exhibit genotoxicity in a dose dependent manner on *Allium* species, concomitant with perturbations in ROS homeostasis. In the present study, a higher and much wider range of NiO-NP concentrations, than those reported by Faisal et al. (2013), were used, since *Allium* species did not show any major perturbationse to lower concentrations of NiO-NP (0.025–2.0 mg L^−1^).

It is known that concentration plays a key role in determining toxic potential of any compound, that can be manifested as having cytotoxic (affecting the cell cycle and cellular organelles) or genotoxic (affecting the genome/DNA) effects, along with their primary and secondary sizes (Gliga et al., 2014). Metal nanoparticles show varied characteristics in aqueous suspensions consequent of the combined effects of structure, surface charge, shape, and size of nanoparticles (Waychunas and Zhang, 2008), along with the interplay of external conditions, like, pH, ionic strength of solution, etc. (Keller et al., 2010). Generally, nanoparticles are predisposed to form aggregates in aqueous medium, mostly owing to increased particle level interaction (Miller et al., 2010; Fairbairn et al., 2011), adversely affecting their surface area and dissolution potential (Baker et al., 2014). This propensity increases with increasing time and concentration of NPs. Therefore, behavior of NiO-NPs in treatment solution was evaluated, through TEM and DLS, to understand the extent of aggregation and secondary size of NiO-NP before exposure (Berne and Pecora, 2000). Physical characterization experiments confirmed that NiO-NP tended to form agglomerates, and this tendency increased in aqueous suspensions, as well as with increasing concentrations, as has been reported in earlier reports for other ENPs too (Hotze et al., 2010). NiO-NP also showed a wide range of PDI in aqueous suspensions; lower the PDI-value, less being the chance of aggregation of the NP in the suspension, as depicted in Table 1. Though the surface charge of NiO-NP was negative (−8.42 ± 3.77 mV), it was observed that this charge was not sufficient enough to sustain the state of suspension when concentration increases, and at higher concentrations NiO-NPs agglomerated and started to sediment down as time lapsed (Ates et al., 2013). Uptake of Ni ions into the plant cell, though dependent on the surface charge and PDI (Clogston and Patri, 2011), constituted a very complex and unique phenomenon altogether. The average size from NiO-NP estimated through DLS (1281 ± 13.97 nm) was much larger than the observed TEM measurements (30–110 nm), because of the aforementioned aggregation that took place in aqueous suspensions (Zhang et al., 2008).

*Allium cepa* root test is the most popular method for evaluation of genotoxicity, mutagenicity, as well as, cytotoxicity of a compound, since the 1940s, because of kinetic characteristics of proliferation and chromosome suitability (Matsumoto et al., 2006; Khanna and Sharma, 2013) and due to close correlation with mammalian systems (Chauhan et al., 1999; Grant, 1999); it is also fast, cost effective and easy to perform (Smaka-Kincl et al., 1996; Çelik and Aslantürk, 2007). Hence this assay was also adopted by the International Program on Plant Bioassays (IPPB) for evaluation of environmental pollutants (Ma and Shelley, 1999). In the present study, a modified version of the *Allium* test was employed, where seedlings of *A. cepa* and four other species of the same genus (*A. schoenoprasum, A. sativum, A. fistulosum*, and *A. porrum*) were used to study potential toxicity of NiO-NP because of their uniformity and ease of study.

*Allium* species readily took up NiO-NP through roots, as evident from the ICP-OES data, that indicated a dose-dependent increase in nickel content in the exposed roots, confirming increased internalization of NiO-NP in treated root tips.

For genotoxicity assessments, CAs signal a sensitive endpoint, which along with MI observations, are useful in identifying environmental chemicals that can affect cytoplasm and nucleus during cell divisions (McLeod, 1969; Topashka-Ancheva et al., 2003; Bakare et al., 2013). These occur upon sudden breaks or exchange of chromosomal materials, and are considered to be of two types, clastogenic and physiological aberrations, both of which can be interpreted as signs of genome instability in the exposed organism. Clastogenic aberrations i.e., MN formation, chromatin bridges, breaks and rings, indicate potential disturbances in DNA or protein synthesis and perturbations of the DNA repair mechanisms (Morita et al., 1991). Physiological aberrations, like C-mitosis, vagrants, stickiness, and laggards to name a few, are indicative of anomalies of chromatin organization or cell cycle fluctuations (Khanna et al., 2016). One particular recurrent trait observed in all the *Allium* species tested was presence of numerous MN in the root tips exposed to lowest doses of NiO-NP suspensions. Micronuclei form due to single stranded DNA breaks, from lagging chromatids or chromatin bridges between anaphases (Utani et al., 2010), are S-phase dependent, and hence are marked indicators of chemical genotoxicity (Fenech et al., 2011). MN-test has come up as the most promising for the evaluation of toxicity and are mostly observed in interphase or prophase (Meng and Zhang, 1992). From the present set of experiments, it may be inferred that NiO-NP has clastogenic effect on dividing root tip cells of *Allium* species, as MN formation was observed in roots exposed to 10 to 500 mg L^−1^ NiO-NP (highest percentage of MN noticed at 125 mg L^−1^ NiO-NP suspension). Another class of chromosomal abnormalities observed included clumped metaphase and chromosomal stickiness found in all the plants upon exposure to lower and intermediate doses of NiO-NP (10–125 mg L^−1^). According to Darlington and Mcleish (1951), stickiness occurs due to degradation, depolymerization or entanglement of inter-chromosomal chromatin fibers whereas disturbed spindle apparatus brings in an irreversible clumped metaphase (Kumari et al., 2009), which also causes anaphase abnormalities and C-mitosis (Al-Ghamery et al., 2000), as are vagrant chromosomes (Rank, 2003). Hence, presence of clumped metaphase and chromosomal stickiness indicate to the large-scale disturbance of spindle fibers in dividing root tips on NiO-NP exposure.

Decrease in mitotic index is a clear indication of arrest in cell growth and is another crucial parameter to study cytotoxicity (Smaka-Kincl et al., 1996). It might be due to inhibition of DNA synthesis or blocking of G2 phase of cell cycle (Sudhakar et al., 2001). In fact, depression of MI-values in exposed plants when compared to their unexposed compatriots, are strong indicators of cytotoxicity and genotoxicity of any test chemical. Hence, depression of MI-values in root tip cells of *Allium* species exposed to NiO-NP clearly indicated cytotoxicity and genotoxicity of this ENP. Taking into consideration the CLV proposed by Sharma and Vig (2012), the present study confirmed that NiO-NP suspensions at concentration higher than 10 mg L^−1^ were sublethal to all species tested, and lethal above 50 mg L^−1^.

It is interesting to note that highest levels of depression of MI and concomitantly highest frequencies of CAs were observed in root tips of all the *Allium* plants exposed to 125 mg L^−1^ NiO-NP suspensions. At higher concentrations of NiO-NP exposure (250–500 mg L^−1^), cell division almost ceased in the meristem of the exposed root tips, probably due to extensive breakdown of cell division machinery, giving rise to pyknosis like state, and also justifying the extreme low MIs recorded, similar to reports of Jones (2000). Binucleate cells that were found in lower concentrations (10–125 mg L^−1^) may have been formed because of interrupted cytokinesis upon NiO-NP exposure, reported earlier by Kalcheva et al. (2009). Reduction of color intensity in the TTC assay and cell viability have close correlation with formation of red formazan generally more prominent at the actively diving meristematic zone of root tips than in the permanent root tissues. In general, lesser formazan was detected in all the *Allium* root tips exposed to different concentrations of NiO-NP, indicating lower cell viability in comparison to unexposed controls. Previous reports by Medley et al. (2008), Song et al. (2009), and Zhu et al. (2010) confirm this observation.

Thus, it is evident that exposure to different concentrations of NiO-NP suspension not only reduced MIs, but also induced clastogenic and physiological aberrations, confirming the mito-depressive character of these ENPs. Significant depression of MI (*p* < 0.005) in exposed root tips of test plants with respect to controls provided definitive proof that NiO-NP can affect cell cycle by inhibiting S-phase, as well as different stages of the division cycle, in a dose-dependent manner, ultimately preventing onset of mitosis and completely destroying the metabolic activities of the cell, as was confirmed by the progressive loss of cell viability in root meristem through TTC assay.

Genotoxic stresses are known to activate intracellular signaling molecules, leading to arrest in growth, DNA repair mechanism activation and/or even cell death (Grishin et al., 2001). Genotoxicity and role of oxidative stress induced cellular death has been elucidated by many workers (Panda et al., 2011; Ghosh et al., 2012). Silver nanoparticles are toxic to both plants and animals (Kumari et al., 2009; Ghosh et al., 2012) and their exposure can induce apoptotic pathway and DNA breaks with a considerable elevation in oxidative stress (Ghosh et al., 2012). TiO_2_, Cd, and Bi (III) NPs have also been shown to manifest genotoxic potential through the oxidative burst mechanism (Kumari et al., 2011; Hossain and Mukherjee, 2013; Liman, 2013; Pakrashi et al., 2014).

It can be mandated from the experiments performed in the present study that treatment of root tips in the sample species evoked generation of ROS significantly when compared to root tips of unexposed control seedlings, and this increased ROS generation was directly attributed to the decrease in vitality of the NiO-NP treated root tip cells as shown by TTC assay. These findings are in agreement with reports of Kumari et al. (2009), Faisal et al. (2013), and Ghosh et al. (2012, 2015), who have also correlated increase in ROS activity upon ENP exposure with cytotoxicity and genotoxicity in exposed animal and plant systems. Lipid peroxidation, detected through accumulation of MDA, formed by converting fatty acids in the cell membrane, is a vital intracellular ROS indicator, and, in the present study each concentration of NiO-NP evoked significant MDA production. Further, upsurge in ROS generation was verified through observing CAT, POD and SOD, which are known components of the antioxidative defense system of plants. A steady relative increase in concentrations of all these enzymes supported the hypothesis that ROS was indeed responsible for the widespread genotoxicity and cytotoxicity observed in this work. This phenomenon is most pronounced at exposure to lower concentrations (10–125 mg L^−1^) of NiO-NP where the cytogenotoxicity is prominent. Interestingly, in the present case study, though conspicuous increase in these marker enzymes at the lower concentrations (10–125 mg L^−1^) of NiO-NP exposure was registered, only marginal rise was seen at higher concentrations (250–500 mg L^−1^). This might be owing to formation of NiO-NP agglomerations in the apoplast and symplast of the cells, though the exact mechanism still not known. Findings from the present set of experiments thus prove that elevated levels of lipid peroxidation and changes in ROS homeostasis as evidenced by alterations in enzymatic profiles in the NiO-NP exposed roots of *A. cepa* can be correlated to the cytogenotoxicity of this ENP.

Catalase, POD, and SOD, the stress marker antioxidant enzymes, respond to biotic or abiotic stress, leading to altered profiles of these enzymes within the cell. In our study too we found that CAT, POD, and SOD showed sharp increase with exposure of NiO-NP to seedlings of all the species of *Allium* studied, in a dose dependent manner. The present findings are in accord with earlier reports (Faisal et al., 2013) that a 24 h-exposure can lead to permanent damage of exposed cells, by wreaking the framework of cell regulation through unprecedented ROS generation, followed by imbalance in antioxidant enzyme systems, irrevocable damage to the chromosome, and possibly the mitochondrial membrane, indicating severe oxidative burst within the cell. Similar mechanism was proposed by earlier workers as possible mechanism of phytotoxicity induced by other ENPs too. Upsurge of ROS within the cell affected the mitochondria, when lipids present in the cellular membranes (including mitochondrial membranes) react with free radicals, leading to formation of lipid peroxides, thus, weakening them. Similar results have already been documented by Premanathan et al. (2011), Zhao et al. (2012), Ghosh et al. (2015), and Ahmed et al. (2016) in both plant and animal systems. Earlier reports by Willekens et al. (1997), Mittler et al. (2004) have conclusively linked rise in CAT and POD levels with oxidative stress. *Phaseolus vulgaris* L. under cadmium and zinc exposure (Chaoui et al., 1997), *Cucumis sativus* L. under high level of salt stress (Zhu et al., 2004), and *Oryza sativa* L. too behaved similarly when subjected to MWCNTs (Tan et al., 2009).

**Figure 10:**
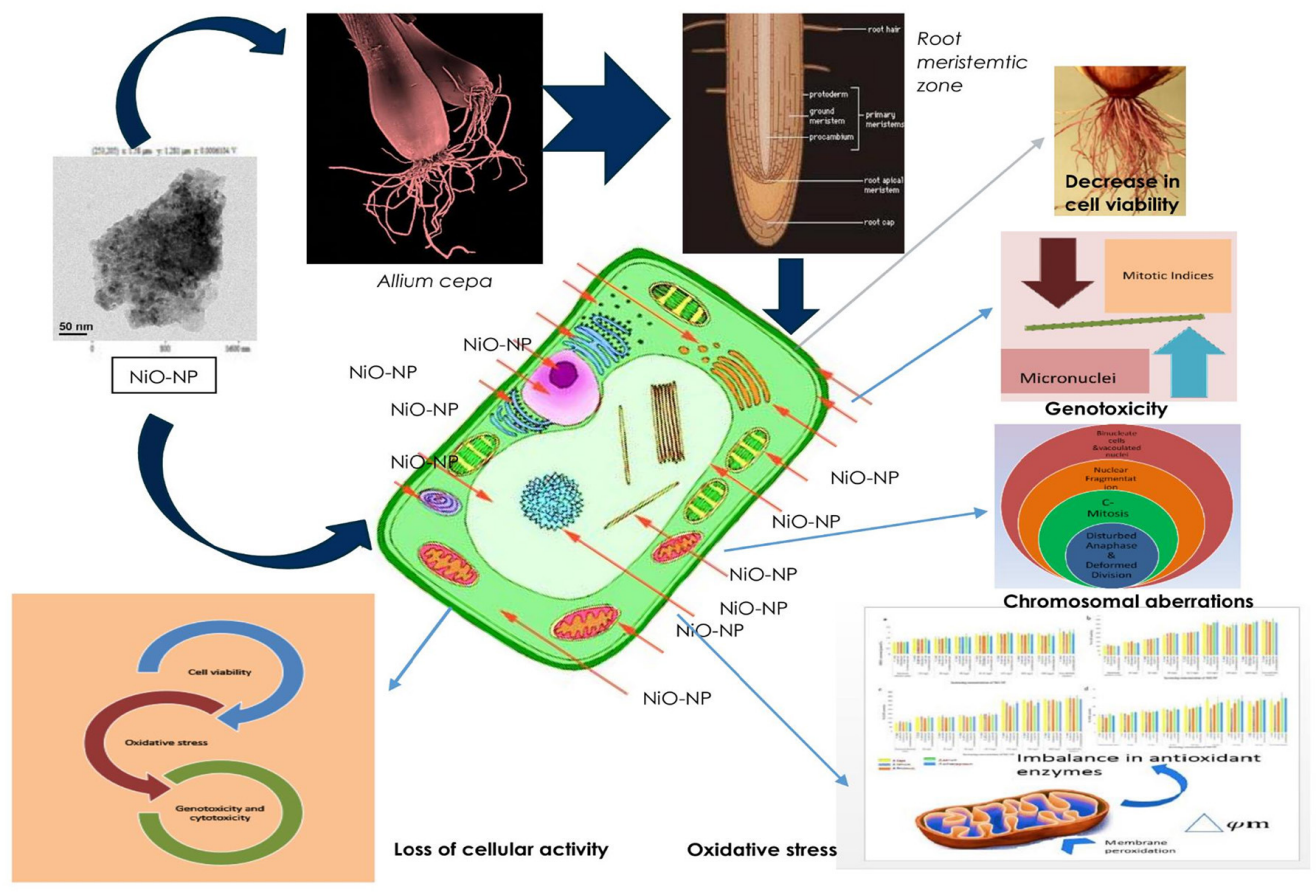
Schematic presentation of possible mechanism of NiO-NP induced cytogenotoxicity in *A. cepa* roots.

Figure 10 sums up the gamut of intracellular events taking place within the root tips of *Allium* species exposed to NiO NP and provides a hypothesis on the possible mechanism by which a cell gets affected owing to NiO-NP exposure.

## Conclusion

The present study showed that NiO-NP is a harbinger of cytogenotoxicity, probably effective through the ROS mediated pathway. NiO-NP interfered with the normal division frequency of cells and induced chromosomes aberrations, thus damaging cell cycle in a dose dependent manner. This study also confirmed induction of genotoxicity at doses as low as 10 mg L^−1^ NiO-NP and exhibited both clastogenic and mito-depressive characteristics, and was cytotoxic as well. The role of ROS mediated induction of cytogenotoxicity was also confirmed. Therefore, further elucidation in unraveling the mechanistic aspect of toxicity induction is needed. However, the present study along with the works done by Faisal et al. (2013) should garner special attention to NiO-NP as a potent environmental pollutant.

## Author contributions

All authors listed have made a substantial, direct, and intellectual contribution to the work, and approved it for publication.

### Conflict of interest statement

The authors declare that the research was conducted in the absence of any commercial or financial relationships that could be construed as a potential conflict of interest.
