# Supplementary material for: Engineered Nickel Oxide Nanoparticle Causes Substantial Physicochemical Perturbation in Plants
Source: Front Chem. 2017 Nov 8;5:92. doi: 10.3389/fchem.2017.00092 (PMC5682307; doi:10.3389/fchem.2017.00092)
Supplement: Supplementary file 1 [file Table1.DOCX]

Supplementary Table 1: table showing decrease in mitotic indices and lethality in a dose dependent manner

| **Plant** | **Treatment** | **Mean MI *** | **Decrease in MI [compared to negative control] (%)** | **Comment**  **(as per Cytotoxic Limit Value)** |
| --- | --- | --- | --- | --- |
| ***A. cepa*** | 10 mgL‾^1^ | 15.33 | 19.599 | **-** |
|  | 25 mgL‾^1^ | 11.42 | **40.105** | **Sub lethal** |
|  | 50 mgL‾^1^ | 8.997 | 52.813 | **lethal** |
|  | 62.5 mgL‾^1^ | 7.57 | 60.297 |  |
|  | 125 mgL‾^1^ | 5.767 | 69.806 |  |
|  | 250 mgL‾^1^ | 5.46 | 71.364 |  |
|  | 500 mgL‾^1^ | 4.68 | 75.454 |  |
|  | 0.4 mM EMS | 3.323 | 82.571 |  |
| 1. ***sativum*** | 10 mgL‾^1^ | 13.567 | **26.13** | **Sub lethal** |
|  | 25 mgL‾^1^ | 9.59 | 47.75 | **lethal** |
|  | 50 mgL‾^1^ | 6.92 | 62.323 |  |
|  | 62.5 mgL‾^1^ | 5.663 | 69.167 |  |
|  | 125 mgL‾^1^ | 3.64 | 80.181 |  |
|  | 250 mgL‾^1^ | 2.92 | 84.101 |  |
|  | 500 mgL‾^1^ | 2.223 | 87.896 |  |
|  | 0.4 mM EMS | 1.783 | 90.292 |  |
| 1. ***fistulosum*** | 10 mgL‾^1^ | 16.567 | **22.9** | **Sub lethal** |
|  | 25 mgL‾^1^ | 12.827 | 40.33 | **lethal** |
|  | 50 mgL‾^1^ | 9.56 | 55.534 |  |
|  | 62.5 mgL‾^1^ | 7.243 | 66.311 |  |
|  | 125 mgL‾^1^ | 5.28 | 75.441 |  |
|  | 250 mgL‾^1^ | 4.193 | 80.497 |  |
|  | 500 mgL‾^1^ | 2.99 | 86.093 |  |
|  | 0.4 mM EMS | 2.48 | 88.465 |  |
| 1. ***porrum*** | 10 mgL‾^1^ | 11.253 | 18.65 | **-** |
|  | 25 mgL‾^1^ | 8.883 | **35.655** | **Sub lethal** |
|  | 50 mgL‾^1^ | 6.38 | 53.878 | **lethal** |
|  | 62.5 mgL‾^1^ | 4.657 | 66.334 |  |
|  | 125 mgL‾^1^ | 2.45 | 82.288 |  |
|  | 250 mgL‾^1^ | 1.227 | 91.129 |  |
|  | 500 mgL‾^1^ | 1.087 | 92.141 |  |
|  | 0.4 mM EMS | 1.427 | 89.684 |  |
| 1. ***schoenoprasum*** | 10 mgL‾^1^ | 15.263 | **22.9** | **Sub lethal** |
|  | 25 mgL‾^1^ | 12.783 | 35.43 | **lethal** |
|  | 50 mgL‾^1^ | 9.353 | 52.767 |  |
|  | 62.5 mgL‾^1^ | 6.493 | 67.207 |  |
|  | 125 mgL‾^1^ | 4.837 | 75.57 |  |
|  | 250 mgL‾^1^ | 2.537 | 87.187 |  |
|  | 500 mgL‾^1^ | 1.657 | 91.631 |  |
|  | 0.4 mM EMS | 1.6 | 91.919 |  |
