# Supplementary figures and images for: Engineered Nickel Oxide Nanoparticle Causes Substantial Physicochemical Perturbation in Plants
Source: Front Chem. 2017 Nov 8;5:92. doi: 10.3389/fchem.2017.00092 (PMC5682307; doi:10.3389/fchem.2017.00092)

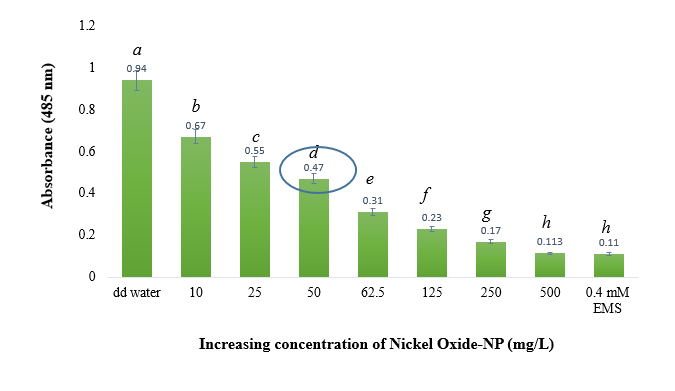

Supplement: Supplementary file 2 [file Image1.TIF]

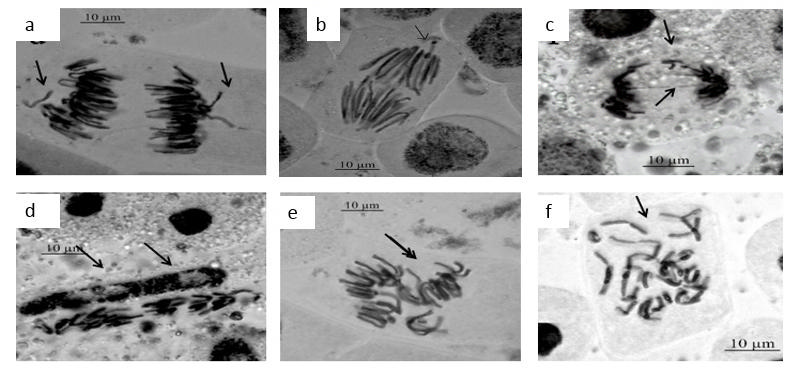

Supplement: Supplementary file 3 [file Image2.TIF]

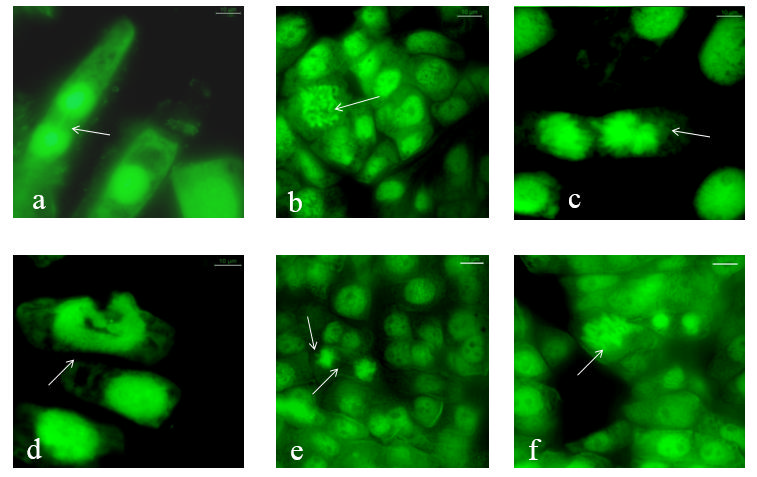

Supplement: Supplementary file 4 [file Image3.TIF]

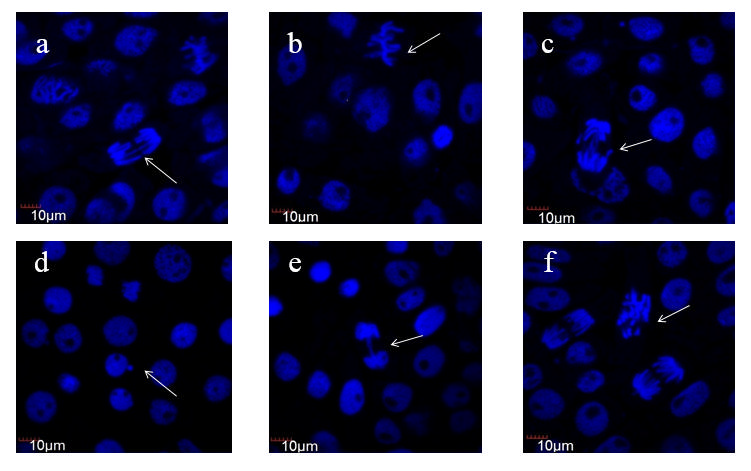

Supplement: Supplementary file 5 [file Image4.TIF]

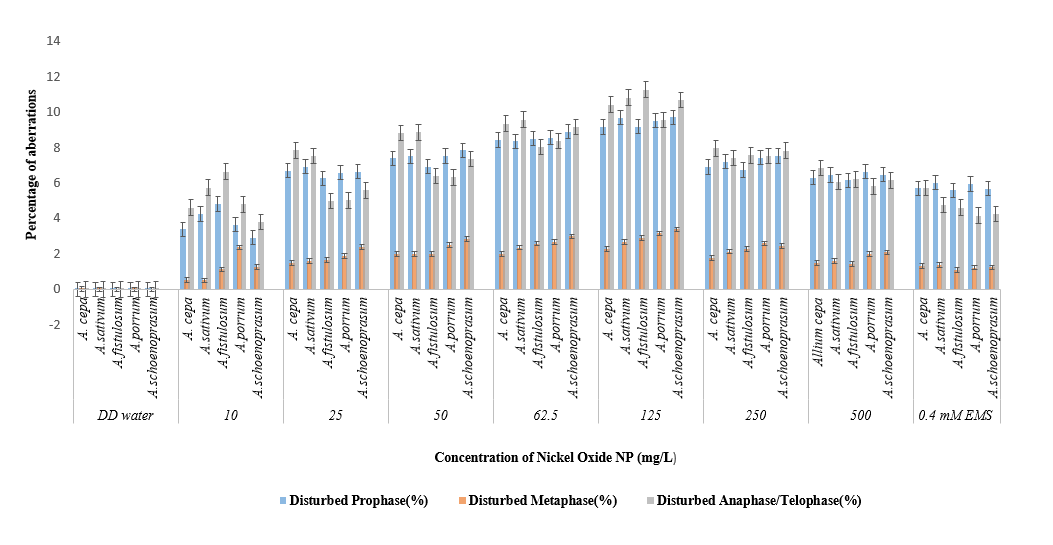

Supplement: Supplementary file 6 [file Image5.TIF]
